# Repeatability of automated measurements by a new anterior segment optical coherence tomographer and biometer and agreement with standard devices

**DOI:** 10.1038/s41598-020-79674-4

**Published:** 2021-01-13

**Authors:** Domenico Schiano-Lomoriello, Kenneth J. Hoffer, Irene Abicca, Giacomo Savini

**Affiliations:** 1 IRCCS – Fondazione Bietti, Rome, Italy; 2 Stein Eye Institute, University of California, Los Angeles, CA USA; 3 St. Mary’s Eye Center, Santa Monica, CA USA

**Keywords:** Diseases, Health care, Medical research

## Abstract

We assess repeatability of automatic measurements of a new anterior segment optical coherence tomographer and biometer (ANTERION) and their agreement with those provided by an anterior segment-optical coherence tomography device combined with Placido-disk corneal topography (MS-39) and a validated optical biometer (IOLMaster 500). A consecutive series of patients underwent three measurements with ANTERION and one with MS-39. A subgroup of patients underwent biometry also with IOLMaster 500. Repeatability was assessed by means of within-subject standard deviation, coefficient of variation (COV), and intraclass correlation coefficient (ICC). Agreement was investigated with the 95% limits of agreement. Paired t-test and Wilcoxon matched-pairs test were performed to compare the measurements of the different devices. Repeatability of ANTERION measurements was high, with ICC > 0.98 for all parameters except astigmatism (0.963); all parameters apart from those related to astigmatism revealed a COV < 1%. Repeatability of astigmatism improved when only eyes whose keratometric astigmatism was higher than 1.0 D were investigated. Most measurements by ANTERION and MS-39 showed good agreement. No significant differences were found between measurements by ANTERION and IOLMaster, but for corneal diameter. ANTERION revealed high repeatability of automatic measurements and good agreement with both MS-39 and IOLMaster for most parameters.

## Introduction

Imaging of the anterior segment has greatly improved in recent years thanks to the introduction of new technologies and the evolution of older ones. Such improvements enable us to obtain unprecedented qualitative information and quantitative measurements of the cornea, anterior chamber, irido-corneal angle, crystalline lens or intraocular lens (IOL).

Optical coherence tomography (OCT), which was initially developed for posterior segment imaging, is now being commonly used also for anterior segment analysis. The first generation of anterior segment optical coherence tomography (AS-OCT), which included instruments such as the Visante (Carl Zeiss Meditec, Germany), used a time-domain system at a central wavelength of 1310 nm but with low axial resolution (18- 25 μm) and a low scan speed (2000 A-scans/second)^1,2^. Those limits were overcome by introducing spectral-domain OCT (SD-OCT) technology, with a higher imaging speed and a better axial resolution^3–5^. Nevertheless, as they used a shorter wavelength light source (around 840 nm), SD-OCT devices had a limited image depth range. Recently, the use of a longer wavelength light source combined with swept-source OCT (SS-OCT) technology has allowed a greater image depth and high-contrast imaging of the entire anterior segment, from the cornea to the posterior surface of the lens^6,7^.

The ANTERION (Heidelberg Engineering, Germany) is a new high-resolution AS-OCT that combines a long wavelength light source with SS-OCT technology^8^. It provides OCT images and information detailing images of the structures from the anterior corneal surface to the posterior lens surface. Moreover, its technology makes it possible to obtain complete biometric measurements of the eye, so that intraocular lens (IOL) power can be calculated.

As with any newly introduced instrument, precision and accuracy are two mandatory requirements. The purpose of this study was to assess the repeatability of automatic measurements of the ANTERION in healthy patients and to assess their agreement with the corresponding parameters provided by an AS-OCT device combined with Placido-disk corneal topography (MS-39, CSO, Florence, Italy) and a validated optical biometer, the IOLMaster 500 (Carl Zeiss Meditec, Germany).

## Results

Ninety-six eyes of 96 patients (mean age = 69.1 ± 16.7 years (range: 19—91) years; females = 58, 60.4%) were enrolled in the repeatability study. Table 1 shows that the repeatability of the ANTERION measurements was high, with ICC > 0.98 for all parameters except astigmatism (0.963) and all parameters apart from those related to astigmatism revealed a COV < 1%.

**Table 1:** Repeatability analysis of the measurements provided by AS-swept source OCT (ANTERION).

Parameter	S_w_	Repeatability	COV (%)	ICC
Sim-K (D)	0.11	0.31	0.26	0.998
Posterior keratometry (D)	0.06	0.17	0.98	0.980
Keratometric astigmatism magnitude (D)	0.16	0.45	16.58	0.986
Keratometric astigmatism axis (D)	16.81	46.55	20.21	0.963
TCP (D)	0.16	0.44	0.36	0.996
TCP astigmatism magnitude (D)	0.13	0.37	12.95	0.990
TCP astigmatism axis (°)	9.01	24.95	9.42	0.993
CCT (µm)	3.89	10.78	0.72	0.996
CD (mm)	0.08	0.22	0.66	0.998
ACD (mm)	0.01	0.03	0.34	1.000
AQD (mm)	0.10	0.27	3.48	0.987
LT (mm)	0.01	0.03	0.33	0.977
AL (mm)	0.01	0.03	0.04	1.000

*Sw* within-subject standard deviation, *COV* coefficient of variation, *ICC* intraclass correlation coefficient, *Sim-K* simulated keratometry, *TCP* total corneal power, *CCT* central corneal thickness, *CD* corneal diameter, *AC* anterior chamber depth, *AQD* aqueous depth, *LT* lens thickness, *AL* axial length.

Astigmatism measurements (magnitude and axis of both keratometric and TCP astigmatism) showed a slightly worse repeatability compared to the other parameters. The repeatability significantly improved when only eyes whose keratometric astigmatism was higher than 1.0 D in all 3 measurements (n = 25, Table 2) were investigated, as the COV decreased below 7%. When the whole sample and the subgroup with keratometric astigmatism > 1.0 D were compared, the F-test confirmed that the difference in variance was statistically significant for the keratometric astigmatism magnitude (variance ratio = 7.31, *p* < 0.001), keratometric astigmatism axis (variance ratio = 28,428.31, *p* < 0.001), TCP astigmatism magnitude (variance ratio = 21.18, *p* < 0.001) and TCP astigmatism axis (variance ratio = 1976.24, *p* < 0.001).

**Table 2:** Repeatability of astigmatism measurements in eyes with keratometric astigmatism > 1.0 D.

Parameter	S_w_	Repeatability	COV (%)	ICC
Keratometric astigmatism magnitude (D)	0.11	0.31	6.27	0.995
Keratometric astigmatism axis (D)	2.11	5.85	2.26	0.999
TCP astigmatism magnitude (D)	0.10	0.28	5.32	0.996
TCP astigmatism axis (°)	2.55	7.07	2.77	0.999

*Sw* within-subject standard deviation, *COV* Coefficient of variation, *ICC* Intraclass correlation coefficient, *TCP* total corneal power.

Table 3 shows the comparison between the measurements of the ANTERION (first scan only) and MS-39. With both devices the mean simulated keratometry was higher than the mean TCP (*p* < 0.0001). Two statistically significant differences were found between the two devices as regards: 1) the posterior keratometry, which was higher (i.e. closer to zero) with the ANTERION, and 2) the magnitude of the TCP astigmatism, which was higher with the MS-39. Low agreement was found for the TCP astigmatism magnitude and axis, as well as for the corneal diameter. The remaining parameters showed good to excellent agreement.

**Table 3:** Comparison between the mean values measured by the ANTERION and by the MS-39.

Parameter	ANTERION	MS-39	95% LoA	*p* value	ICC
	Mean ± SD	Mean ± SD			
Sim-K (D)	43.95 ± 1.51	43.98 ± 1.50	– 0.68 to + 0.62	n.s	0.976
Posterior keratometry (D)	– 6.18 ± 0.23	– 6.47 ± 0.24	+ 0.14 to + 0.41	< 0.0001	0.556
TCP (D)	43.47 ± 1.57	43.44 ± 1.55	– 0.78 to + 0.84	n.s	0.965
TCP astigmatism magnitude (D)	1.01 ± 0.79	1.23 ± 0.91	– 1.50 to + 1.00	0.0013	0.699
TCP astigmatism axis (°)	98.67 ± 64.91	103.98 ± 70.18	– 71.3 to + 60.7	n.s	0.869
CCT (µm)	538.59 ± 33.93	537.98 ± 33.17	– 9.9 to + 11.11	n.s	0.969
CD (mm)	11.83 ± 0.44	11.69 ± 0.64	– 0.89 to + 1.16	n.s	0.752
AQD (mm)	2.76 ± 0.49	2.80 ± 0.44	– 0.44 to + 0.36	n.s	0.898

*LoA* limits of agreement, *ICC* Intraclass correlation coefficient (between ANTERION and MS-39), *Sim-K* simulated keratometry, *TCP* total corneal power, *CCT* central corneal thickness, *CD* corneal diameter, *AQD* aqueous depth.

A subgroup of fifty-seven eyes of 57 patients scheduled for cataract surgery (mean age = 75.2 ± 6.1 years (range: 60—88 years); females = 36, 63.2%) underwent optical biometry with both the ANTERION and IOLMaster 500, which showed a high rate of measurement success (55 eyes out of 57, 96.5%, with each device). Overall, the examination was successful with both instruments in 53 eyes (mean age = 75.5 ± 6.1 years, range: 60 – 88 years; females = 34, 64.2%). Table 4 reports the mean values and their agreement.

**Table 4:** Comparison between the mean values measured by the ANTERION and IOLMaster 500.

Parameter	ANTERION	IOLMaster 500	95% LoA	*p* value	ICC
	Mean ± SD	Mean ± SD			
AL (mm)	23.62 ± 1.63	23.63 ± 1.62*	– 0.06 to + 0.05	< 0.0001	1.000
ACD (mm)	3.21 ± 0.43	3.17 ± 0.43	– 0.50 to + 0.57	n.s	0.888
Sim-K (D)	43.85 ± 1.53	43.84 ± 1.58	– 0.68 to + 0.70	n.s	0.987
CD (mm)	11.83 ± 0.46	12.04 ± 0.39	– 0.72 to + 0.31	< 0.0001	0.839

*SD* standard deviation, *LoA* limits of agreement, *ICC* Intraclass correlation coefficient (between ANTERION and IOLMaster 500), *AL* axial length, *ACD* anterior chamber depth, *Sim-K* simulated keratometry, *CD* corneal diameter.

## Discussion

The application of SS-OCT technology to anterior segment tomography and biometry has reduced the time of image acquisition and improved axial and lateral resolution and tissue penetration^8,9^. The aim of our study was to investigate the measurements provided by one of the latest SS-OCT optical biometers, the ANTERION, in order to assess its repeatability as well as its agreement with an AS-OCT and with a validated traditional optical biometer.

The precision of anterior segment measurements is crucial when planning refractive or cataract surgery and in order to evaluate the progression of corneal diseases, such as keratoconus.

Our data show a high repeatability of the measurements obtained with the ANTERION, as the values showed an ICC > 0.9. Our results about repeatability are in line with those recently published^10^. As previously reported for other devices^11–13^, the repeatability was slightly lower for astigmatism magnitude and axis. However, if we consider the subgroup including only eyes with a keratometric astigmatism higher than 1 D, the repeatability improved considerably. This is important, because keratometric astigmatism measurement is used to calculate the cylinder of toric IOLs, which are usually implanted in eyes with astigmatism higher than 1 D.

In our study we also compared the agreement between ANTERION measurements and the corresponding parameters measured with the MS-39, which has already been found to achieve high repeatability^3–14^. Most measurements (Sim-K, TCP, CCT, CD and AQD) revealed good agreement without statistically significant differences. However, a statistically significant difference and a lower agreement were found for posterior keratometry and TCP astigmatism magnitude measurements. While the difference in the case of the former parameter seems clinically negligible, the latter may have an impact for toric intraocular lens (IOL) calculations and therefore deserves future studies in this context. Differences between tomographer measurements of total corneal astigmatism have already been reported and may be related to the different technologies they are based on^15^.

The comparison with the IOLMaster also revealed good agreement for all parameters. Measurements of AL were significantly different from a statistical, but not clinical point of view, with a mean discrepancy of just 0.01 mm, and agreement was high. These results, which are consistent with those previously reported in a study comparing the IOLMaster with the LenStar LS900 (Haag-Streit)^16^, may be explained by the fact that both the ANTERION and the IOLMaster use the group refractive index to convert the optical path length into a physical AL, as recommended by Haigis in 1999^9^. This is the reason why agreement for AL measurements by different optical biometers has always been reported to be high^7,17–19^.

In our study, measurement of AL using the ANTERION had a high rate of success (96.5%), in line with previous studies that have described a range from 92 to 100% for different SS-OCT devices^20–22^ In contrast with the literature, which has reported a lower success rate in AL measurement using the IOLMaster 500^21,23^, our data showed the same success rate for the ANTERION and IOLMaster 500. As an explanation, the authors suggest the fact that the group of patients in this study did not include opaque cataracts.

As regards ACD measurements, our data must be interpreted with caution, since the current version of ANTERION software does not automatically supply this parameter, which we calculated by adding the measured AQD to CCT. Previous studies have found that the IOLMaster 500, which measures ACD through a lateral source of light, usually provides lower mean values compared to other optical biometers^16,24–31^. Future studies will need to assess whether ACD measurements provided automatically by the ANTERION (when commercially available) are close to those of other optical biometers.

Some limitations of our study should be considered. We excluded pathological eyes and postoperatively altered corneas, which deserve further investigations. Moreover, we did not assess the accuracy of ANTERION measurements for IOL power calculation; however, a study on this subject is already under way.

In conclusion, based on the findings of our study, the measurements provided by the new SS-OCT ANTERION show high repeatability and good agreement with those of the MS-39 for anterior segment parameters and with the IOLMaster 500 for biometric parameters.

This means that most measurements by the ANTERION and MS-39 can be used interchangeably in clinical practice and that the formula constants optimized for the IOLMaster 500 are likely to be valid for the ANTERION too.

## Materials and methods

### Participants

In this prospective study, we included a consecutive series of patients examined at the Anterior Segment Unit of G.B. Bietti Foundation IRCCS, Rome, Italy between October 2019 and January 2020. Subjects aged ≥ 18 years and without a history of ocular surgery, contact lens wear in the last month, corneal disease or trauma were eligible for enrollment. All patients underwent a complete ophthalmic evaluation, including distance-corrected visual acuity measurement, anterior segment biomicroscopy, Goldmann applanation tonometry and fundus biomicroscopy. For the purposes of this study, anterior segment tomography and optical biometry were performed in all subjects using the ANTERION Cataract App. Anterior segment tomography was performed in all eyes with the MS-39. Finally, a subgroup of patients scheduled for cataract surgery underwent optical biometry also with the IOLMaster 500.

We excluded patients with ocular diseases that could affect fixation and the quality of acquisition, such as corneal opacity or any maculopathy. Other exclusion criteria were the presence of keratoconus or suspect keratoconus, as shown by corneal tomography with the MS-39, and contact lens use in the past month. All research procedures described in this work adhered to the tenets of the Declaration of Helsinki.

All recruited subjects gave written informed consent. The study protocol was approved by the Ethics Committee of G.B. Bietti Foundation.

### Instruments

The ANTERION is a swept-source AS-OCT with a 1300 nm wavelength light source, with a speed of 50,000 A-scans/second. It images the anterior segment of the eye with an axial depth of 14 mm, a lateral width of 16.5 mm, an in tissue axial resolution of < 10 µm and lateral resolution of 30–45 mm^8^. All measurements are assisted by an eye-tracking technology, centered on the corneal vertex. Corneal information is based on 65 radial scans, generating data for corneal maps and analysis at 8 mm diameter. The Cataract App acquisition was used to acquire the data (software version 1.2) and the following parameters were analyzed:

- Simulated keratometry (Sim-K) average: this is defined as the average corneal power calculated at a 3 mm ring, based on anterior corneal axial curvature. The conversion of anterior radii to keratometry values is performed using the standard keratometric index of 1.3375.
- Keratometric astigmatism: this is defined as the dioptric difference between the steep and the flat corneal meridians, as calculated by Sim-K.
- Posterior keratometry average: this is defined as the posterior corneal power calculated at a 3 mm ring, based on the posterior corneal axial curvature. The curvature of posterior radii to keratometry values is performed using the refractive indices of the cornea (1.376) and aqueous (1.336).
- Total corneal power (TCP) average: this is defined as the average refractive power of the cornea, calculated by ray tracing at a 3 mm ring through both corneal surfaces. Ray tracing determines how parallel light beams are refracted according to the slope of the cornea, the true refractive indices (n_cornea_ = 1.376, n_aqueous_ = 1.336), and the exact point of refraction.
- TCP astigmatism: this is defined as the dioptric difference between the steep and the flat corneal meridians, as calculated on the basis of TCP.
- Central corneal thickness (CCT): this is defined as the perpendicular distance between the central anterior and posterior corneal surfaces, measured from the anterior corneal vertex.
- Corneal diameter (CD): this parameter, which is defined as white-to-white on the instrument, is measured as the horizontal distance between the nasal and temporal limbus, as obtained on the camera image.
- Aqueous depth (AQD): this is defined as the distance from the central posterior corneal surface to the central anterior lens surface, measured perpendicular to the anterior corneal surface and along the line of sight. By adding AQD and CCT it is possible to calculate the anterior chamber depth (ACD) as the distance from the corneal epithelium to the anterior lens surface as used in IOL power formulas.
- Lens thickness (LT): this is defined as the distance between the central anterior and posterior lens surfaces, measured perpendicular to the anterior corneal surface;
- Axial length (AL): this is defined as the distance between the anterior corneal surface and the retinal pigment epithelium, along the line of sight. AL is calculated by means of a group refractive index.

The MS-39 (software version 3.6) is a spectral-domain OCT combined with a Placido disk. It acquires one Placido top-view image and a series of 25 SD-OCT radial scans at a wavelength of 840 nm, with an axial resolution of 3.5 µm in tissue, a lateral resolution of 35 µm and a depth of 7.5 mm. Data for the anterior surface from the Placido image and SD-OCT scans are merged using a proprietary method. High repeatability of its measurements has been previously reported^3^.

The IOLMaster 500 (software version 5.4) is a first generation optical biometer using partial coherence interferometry (PCI) for AL measurement. The keratometry value is measured by means of a six-point telecentric reflectometry technique in an approximately 2.5 mm diameter pattern, while ACD is measured by lateral slit beam illumination. Several articles have confirmed the high repeatability of its measurements^32–35^.

### Measurement procedures

One eye of each patient was randomly selected. ANTERION and MS-39 scans were taken on the same day, between 11 A.M. and 5 P.M., to minimize diurnal changes. Examinations were performed without pupil dilation in a dimly lit room, after each instrument had been calibrated at the beginning of the day. In order to assess the repeatability of ANTERION measurements, the tomographic and biometric parameters were acquired 3 times by an experienced examiner (MR). After each measurement, the patient was invited to sit back and the device was realigned before the subsequent scan.

With the IOLMaster 500, the measurements were acquired only once if the scan quality, as indicated by the instrument, was good; otherwise, measurements were repeated until a good quality was achieved.

### Statistical analysis

The repeatability analysis was carried out according to the standards of the International Organization for Standardization^36^. The following parameters were investigated:

1. Pooled within-subject standard deviation (S_w_) and intra-session test–retest variability (also known as repeatability or limits of repeatability). The latter was obtained by multiplying the S_w_ by 2.77^37^. Test–retest repeatability indicates an estimate of the limits within which 95% of measurements for the same subject should be.
2. Coefficient of variation (COV). This was calculated as the S_w_ divided by the mean of the measurements and expressed as a percentage^38^.
3. Intraclass correlation coefficient (ICC). This is the ratio of the between-subjects variance to the sum of the S_w_ and the between-subjects variance. The ICC, which is close to 1.0 when there is no variance between repeated measurements, was automatically calculated using the 2-way mixed model and absolute agreement. ICCs ranging from 0 to 1 are classified as follows: ICC less than 0.75 = poor agreement; ICC 0.75 to less than 0.90 = moderate agreement and ICC 0.90 and more = high agreement^39^.

The variance of different measurements was compared by means of the F-test.

Based on the paper by McAlinden^40^, a minimum sample size of 96 eyes was estimated to be necessary to assess repeatability: this sample size allowed us to have a 0.10 confidence in the estimate with 3 repeated measures.

Agreement among the measurements provided by the different devices was assessed by means of the 95% limits of agreement (LoA)^41^. The difference between measurements (y-axis) is plotted against their means (x-axis). The mean difference is the estimated bias and the standard deviation (SD) of the differences shows the fluctuation around the mean. The 95% LoAs are calculated as the mean ± 1.96 SD of the differences between the 2 measurement techniques. The sample size for the agreement between ANTERION biometry and IOLMaster was calculated, according the recommendation of McAlinden^42^ using the following approximated formula: 1.96 $\frac{{\sqrt {3s}^{2} }}{\surd n} =$ desired confidence of LoA.

We speculated that the desired confidence of LoA for axial length was 0.02 with a standard deviation of mean difference (s) 0.04, which was not clinically important.

We also calculated the ICC to complete our analysis of agreement between the instruments (ANTERION versus MS-39 and versus IOLMaster 500, respectively).

In addition, Statistics were analyzed by means of MedCalc (version 12.3.0.0, MedCalc Software Ltd, Ostend, Belgium) and Instat (version 3.10, Graphpad Software Inc, La Jolla, CA).

## Data Availability

The datasets used and analysed to support the findings of this study are available from the corresponding author upon request.
